# Screening for PPAR Responsive Regulatory Modules in Cancer

**DOI:** 10.1155/2008/749073

**Published:** 2008-06-05

**Authors:** Merja Heinäniemi, Carsten Carlberg

**Affiliations:** Life Sciences Research Unit, University of Luxembourg, 1511 Luxembourg, Luxembourg

## Abstract

Peroxisome proliferator-activated receptors (PPARs) have via their large set of target genes a critical impact on numerous diseases including cancer. Cancer development involves numerous regulatory cascades that drive the progression of the malignancy of the cells. On a genomic level, these pathways converge on regulatory modules, some of which contain colocalizing PPAR binding sites (PPREs). We developed an in silico screening method that incorporates experiment- and informatics-derived evidence for a more reliable prediction of PPREs and PPAR target genes. This method is based on DNA-binding data of PPAR subtypes to a panel of DR1-type PPREs and tracking the enrichment of binding sites from multiple species. The ability of PPAR*γ* to induce cellular differentiation and the existence of FDA-approved PPAR*γ* agonists encourage the exploration of possibilities to activate or inactivate PPRE containing modules to arrest cancer progression. Recent advances in genomic techniques combined with computational analysis of binding modules are discussed in the review with the example of our recent screen for PPREs on human chromosome 19.

## 1. INTRODUCTION

Cellular proliferation and differentiation are controlled by transcriptional regulation of a large subset of the human genome. The transcriptomes of normal and tumor cells as revealed by microarray analysis show significant differences [1] suggesting that in cancer the precise transcriptional control got lost due to overactive oncogenes and loss of function of tumor suppressor genes, many of which are coding for transcription factors. For a molecular insight into cancer, the transcriptional regulation of probably thousands of genes has to be uncovered in detail by integrating expression array data with regulatory site location data [2]. Although the understanding of the regulation of a couple of key genes, like the cyclin-dependent kinase inhibitor *p*21^*WAFI*/*CIPI*^ [3], is already quite advanced, for the majority of the cancer-associated genes such detailed analyses have not been performed. Even “big biology” projects, such as ENCODE [4], have focused only on 1% of the human genome sequence so far, while other genome-wide scans, for example, for histone modifications [5–7] or transcription factor binding [8, 9], had to concentrate on only a subset of modifications and factors under limited experimental conditions. Databases, such as oncomine [1] for gene expression data and the UCSC genome browser [10] for visualization of genome-wide chromatin immunoprecipitation data and transcription factor binding site location data, allow the combination of data from various projects. Together, these data resources may provide sufficient insight to understand the regulation of an individual gene in a complex disease state, such as cancer. In addition, efforts to improve bioinformatics methods predicting the binding and interaction of transcription factors together with more extensive experimental datasets will fill important gaps [11].

Each individual gene is under the control of a large set of transcription factors that can bind upstream and downstream of its transcription start site (TSS) [12]. These sites typically arrange into collections of neighboring sites, the so-called modules or enhancers. Modules of transcription factors that act on focused genomic regions have been shown to be far more effective than individual factors on isolated locations and can act from large distances up to hundreds of thousands of base pairs. In an ideal case such transcription factor modules can be identified by parallel and comparative analysis of their binding sites. Here, bioinformatics approaches can be of great help, in case they can predict the actions of the transcription factors precisely enough [13].

PPARs are transcription factors that have the special property to be ligand-inducible, which they share with most other members of the nuclear receptor superfamily [14]. This property has attracted a lot of interest in the nuclear receptor family as possible therapeutical targets in context of cancer. PPARs were initially described as the nuclear receptors for compounds that induce peroxisome proliferation in rodents [15], but now they are know to be important sensors of cellular levels of fatty acids and fatty-acid derivatives that are mainly derived from the lipoxygenase and cyclooxygenase pathways [16]. Polyunsaturated fatty acids activate the three PPAR subtypes with relatively low affinity, whereas fatty acid derivatives show more binding selectivity [17]. PPARs are prominent players in the metabolic syndrome because of their role as important regulators of lipid storage and catabolism [18], but they also regulate cellular growth and differentiation and therefore have an impact on hyperproliferative diseases, such as cancer [19]. Bioinformatic approaches to identify genomic targets of PPARs and important cancer regulatory modules with colocalizing PPREs, as they will be described below, should have a major impact on understanding the role and potential therapeutic value of PPARs in cancer.

## 2. THE PPAR SUBFAMILY

The three PPAR subtypes *α* (NR1C1), *β*/*δ* (NR1C2), and *γ* (NR1C3) are coexpressed in numerous cell types from either ectodermal, mesodermal, or endodermal origin, although their concentration relative to each other varies widely [20, 21]. Importantly, most tumor cells express at least one PPAR subtype at higher levels suggesting that PPAR ligands may modulate the transcription of many PPAR target genes in a beneficial way.

PPAR*α* is highly expressed in cells that have active fatty acid oxidation capacity including hepatocytes, cardiomyocytes, enterocytes, and the proximal tubule cells of the kidney [22]. This PPAR subtype is a central regulator of hepatic fatty acid catabolism and glucose metabolism. Furthermore, it potently represses the hepatic inflammatory response by downregulating the expression of numerous genes, such as various acute-phase proteins. PPAR*α* is the molecular target for the hypolipidemic fibrates, a group of drugs that are prescribed for their ability to lower plasma triacylglycerols and elevate plasma HDL (high-density lipoprotein) levels.

PPAR*β*/*δ* is expressed ubiquitously and often displays at higher expression levels than PPAR*α* and *γ*. It stimulates fatty acid oxidation in both adipose tissue and skeletal muscle, regulates hepatic VLDL (very low-density lipoprotein) production and catabolism and is involved in wound healing by governing keratinocyte differentiation [23].

PPAR*γ* is expressed predominantly in adipose tissue and the immune system and exists as two distinct protein forms *γ*1 and *γ*2, which arise by differential TSSs and alternative splicing [22]. PPAR*γ* is the master regulator of adipogenesis and regulates cell-cycle withdrawal, as well as induction of fat-specific target genes that are involved in adipocyte metabolism [24]. PPAR*γ* stimulates the expression of numerous genes that are involved in lipogenesis, including those for adipocyte fatty acid-binding protein, lipoprotein lipase, and fatty acid translocase (CD36). The general role for PPAR*γ* in the regulation of lipid metabolism is underlined by the therapeutic utilization of the PPAR*γ* ligands thiazolidinediones in obesity-linked type II diabetes [25].

## 3. PPARs AND THE TRANSCRIPTIONAL MACHINERY

An essential prerequisite for the direct modulation of transcription by PPAR ligands is the location of at least one activated PPAR protein close to the TSS of the respective primary PPAR target gene. This is commonly achieved through the specific binding of PPARs to a DNA binding site, the so-called PPRE, and DNA-looping towards the TSS [26]. In detail, the DNA-binding domain of PPARs contact the major groove of a double-stranded hexameric DNA sequence with the optimal AGGTCA core binding sequence. PPARs bind to DNA as heterodimers with the nuclear receptor retinoid X-receptor (RXR) [27]. PPREs are therefore formed by two hexameric core binding motifs in a direct repeat orientation with an optimal spacing of one nucleotide (DR1), where PPAR occupies the 5′-motif [28]. However, characterization of PPREs from regulated gene promoters has resulted in a large collection of PPREs that deviate significantly from this consensus sequence. An extensive binding data collection for PPARs was recently published [29], where more critical deviations and well-tolerated deviations from the consensus were identified as will be further explained in the following chapters.

When a nuclear receptor, such as PPAR, is bound to PPREs in the regulatory regions of its target genes, it recruits positive and negative coregulatory proteins, referred to as coactivators [30] and corepressors [31], respectively. In consequence, the transcriptional output is dependent on cell- and time-specific expression patterns of these coregulators and can produce distinct modulations of transcription factors, such as PPARs, due to differences in the relative corepressor and coactivator protein levels. This aspect has diagnostic and therapeutic value and can be extracted from expression level data in different types of cancer [32]. Most unliganded nuclear receptors preferentially interact with corepressors to mediate repression, but PPARs have been found to show a reasonable level of constitutive activity [33], that is, in the absence of ligand coactivator proteins can compete for binding. Most coregulators are not exclusive to PPARs and even not specific to nuclear receptors, but are also used in a similar manner by other transcription factors [34]. One group of coregulators covalently modifies histone proteins, which are as nucleosome constituents the main chromatin proteins. This acetylation/deacetylation and methylation/demethylation follows a precise and combinatorial code, the so-called histone code [35]. The second group of coregulators includes ATP-dependent chromatin remodeling factors that modulate the accessibility of genomic regions to transcription factors and to the basal transcriptional machinery [36]. Recently, their actions have been monitored on genome-wide level to reveal common patterns of transcriptionally active regions and regulatory sites [5, 7, 9]. These snap-shots have provided important insights to common regulatory code, whereas more detailed studies have explored the dynamics of these processes as described below.

Repression and activation are more likely achieved by a series of sequential events that are mediated by multiple enzymatic activities that are promoter and cell-type specific. Transcriptional regulation is a highly dynamic event of rapid association and dissociation of proteins and their modification, including degradation and de novo synthesis. A pattern of recruitment and release of cohorts of coregulatory complexes was demonstrated on a single region of the *trefoil factor-1* promoter in breast cancer cells [37]. This study revealed detailed and coordinated patterns of coregulator recruitment and preferential selectivity for factors that have similar enzymatic activities. Similar cycling was also observed for the recruitment of PPAR*β*/*δ* to the TSS of the *pyruvate dehydrogenase kinase* 4 (Degenhardt et al., unpublished). Understanding the events that lead to the disturbance of such coordinated action of regulatory proteins in cancer progression could help finding means to reinitiate the coordinated regulation. Partial restoration of regulation was demonstrated on the *trefoil factor-1* promoter by removal of methylation in an unresponsive cell line [38].

## 4. PPARs IN CANCER

The rapid growth of tumor cells is highly dependent on the availability of macronutrients and their metabolism. In their role as master regulators of lipid metabolism, all three PPAR subtypes have at least an indirect function in controlling cellular growth [26]. Moreover, the dominant function of PPAR*γ* in adipocyte differentiation and the suppression of apoptosis in keratinocytes by PPAR*β*/*δ* suggest a direct role of PPARs in the control of cellular growth and death [19]. As a consequence, a number of prominent PPAR target genes, such as *angiopoietin-like 4*, *lipoprotein lipase*, *LDL-receptor-related protein 1*, and *caveolin-1*, were described to be involved in the control of tumor cell growth [39–42]. Furthermore, there is a strong physiological link between chronic inflammation and the onset of cancer [43]. In this way, the anti-inflammatory actions of PPARs [44] provide an additional argument for their control function on cellular proliferation, differentiation, and apoptosis.

However, there is also evidence to state that PPARs may in some cases promote cancer progression. PPAR*β*/*δ* has been implicated in colorectal carcinogenesis [45], its mRNA is often upregulated in tumors and the deletion of the PPAR*β*/*δ* gene results in a profound loss of tumorigenicity in nude mice [46]. Moreover, PPAR*β*/*δ* was found to have an essential role in constraining tumor endothelial cell proliferation to allow the formation of functional tumor microvessels, that is, the receptor is important for angiogenesis [47].

As a general argument, we can propose that the main role of PPARs, the control of metabolism or inflammation, may also contribute to the regulation of cellular growth. How that translates (via transcriptional regulation) into interference in cancer progression or change to a more benign phenotype, may be highly dependent on cancer type and state. In fact, the net effect of the activation of some PPAR target genes may rather result in the stimulation of cellular proliferation than in its inhibition, when examined alone. Data on gene expression, on regulatory modules, on their accessibility, and on the binding of PPARs to those modules need to be joined, in order to get a handle on the pleiotropic effects of PPARs in cancer.

## 5. METHODS FOR IN SILICO SCREENING OF TRANSCRIPTION FACTOR BINDING SITES

The specificity of PPARs for their binding sites allows constructing a model to describe the PPRE properties that can be used to predict potential binding sites in genomic sequences. For this, the PPAR binding preference, often expressed as position weight matrix (PWM), has to be described on the basis of experimental data, such as series of gel shift assays with a large number of natural binding sites [48–51]. However, PPAR-RXR heterodimers do not only recognize a pair of the consensus binding motifs AGGTCA, but also a number of variations to it. Dependent of the individual PWM description, this leads to a prediction of PPREs every 1000 to 10000 bp of genomic sequence. This probably contains many false positive predictions, which is mainly due to scoring methodology and the limitations that are imposed by the available experimental data. For example, the quantitative characteristics of a transcription factor, that is, its relative binding strength to a number of different binding sites, is neglected in a position frequency matrix, where simply the total number of observations of each nucleotide is recorded for each position. Moreover, in the past there was a positional bias of transcription factor binding sites upstream in close vicinity to the TSS. This would be apparent from the collection of identified PPREs, but is in contrast with a multigenome comparison of nuclear receptor binding site distribution [52] and other reports on wide-range associations of distal regulatory sites [7].

Internet-based software tools, such as TRANSFAC [53], screen DNA sequences with databases of matrix models. One approach used PWMs to describe the binding preferences of PPARs using all published PPREs [54]. The accuracy of such methods can be improved by taking the evolutionary conservation of the binding site and that of the flanking genomic region into account. Moreover, cooperative interactions between transcription factors, that is, regulatory modules, can be taken into account by screening for binding site clusters. The combination of phylogenetic footprinting and PWM searches applied to orthologous human and mouse gene sequences reduces the rate of false predictions by an order of magnitude, but leads to some reduction in sensitivity [11]. Recent studies suggest that a surprisingly large fraction of regulatory sites may not be conserved but yet are functional, which suggests that sequence conservation revealed by alignments may not capture some relevant regulatory regions [55].

In effect, these approaches and tools are still insufficient and there has to be a focus on the creation of bioinformatics resources that include more directly the biochemical restrains to regulate gene transcription. One important aspect is that most putative transcription factor binding sites are covered by nucleosomes, so that they are not accessible to the transcription factor. This repressive environment is found in particular for those sequences that are either contained within interspersed sequences, are located isolated from transcription factor modules, or lie outside of insulator sequences marking the border of chromatin loops [56]. This perspective strongly discourages the idea that isolated, simple PPREs may be functional in vivo. In turn, this idea implies that the more transcription factor binding sites a given promoter region contains and the more of these transcription factors are expressed, the higher is the chance that this area of the promoter becomes locally decondensed.

The PAZAR information mall [57] is a tertiary database that is build on the resource of a multitude of secondary databases and provides a computing infrastructure for the creation, maintenance, and dissemination of regulatory sequence annotation. The unambiguous identification of the chromosome location for any given transcription factor binding site using genomic coordinates allows to link the results from “big biology” projects, such as ENCODE [4], and other whole genome scans for histone modification and transcription factor association. Unfortunately, so far only a few boutiques have been opened inside the PAZAR framework. In order to benefit from binding site predictions, it is still necessary to explore dedicated resources. For example, the well-known regulator of cell cycle progression, the transcription factor p53, has an own dedicated database (p53FamTaG) for integration of gene expression and binding site data [58].

The concept of cancer-specific regulatory modules has raised increasing attention recently. Genome-wide prediction of enhancers based on analysis of transcription factor binding affinity by a computational tool, called enhancer element locator [8], was shown effective to dissect which types of cancer can be targeted by a given transcription factor. Predictions validated in transgenic mouse embryos revealed the presence of multiple tissue-specific enhancers in mouse *c-myc* and *N-myc* genes, which has implications for organ-specific growth control and tumor-type specificity of oncogenes.

## 6. THE CLASSIFIER METHOD FOR PPREs

Approaches for PPRE predictions have been based on a collection of disparate binding data. To combine evidence from several publications for an efficient binding model has challenges thus creating a demand for a coherent binding dataset. The recently published classifier method [29] used the in vitro binding preferences of the three PPAR subtypes on a panel of 39 systematic single nucleotide variations of the consensus DR1-type PPRE (**AGGTCA**A**AGGTCA**) [59] as an experimental dataset. The single nucleotide variants were sorted into three classes, where in class I the PPAR subtypes are able to bind the sequence with a strength of 75 ± 15% of that of the consensus PPRE, in class II with 45 ± 15%, and in class III with 15 ± 15%. Although the overall binding pattern of the three PPAR subtypes showed no major differences, some variations gave rise to a PPAR subtype-specific classification. Additional 130 DR1-type PPREs were sorted on the basis of counting increasing number of variations from the consensus and taking into account the single nucleotide variant binding strength. Those variants that alone decrease the binding only modestly (class I) could be combined with even three deviations from consensus still resulting in more than 20% binding relative to consensus. Other combinations resulted in faster loss of binding detailed in 11 categories, where such combinations still resulted in more than 1% relative binding.

**Figure 1:**
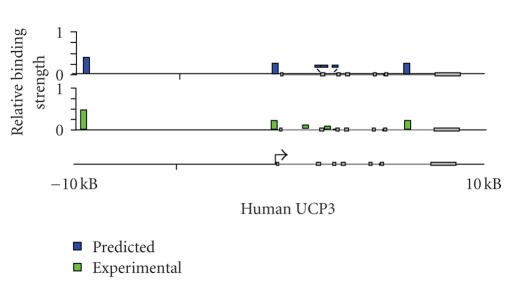
Comparison of in silico and experimental analysis of PPAR target genes. Overview of the genomic organization of the *UCP3* gene; 10 kB upstream and downstream of its TSS are shown (horizontal black line). Putative PPREs were identified using the classifier method performing in silico screening of the genomic sequences. For each predicted PPRE, the calculated binding strength of PPAR*γ* is represented by column height. The average in vitro DNA binding strength of PPAR*γ*-RXR heterodimers was also determined by gel shift experiments.

The in silico binding strength predictions of PPAR-RXR heterodimers were confirmed by gel shift assays for the six PPREs of the *uncoupling protein 3* (*UCP3*) gene and showed a deviation of less than 15% (Figure 1). Moreover, from 23 investigated genomic regions that were selected from eight genes, 17 regions display significant inducibility in the presence of PPAR ligands and in living cells. PPAR*α* and RXR*α* associated with 16 of these regions. For the *UCP3* gene, for which previously no regulatory regions had been described to account for the effect of PPAR ligands on its mRNA transcription, three functional areas were identified [29].

The main advantage, when comparing the classifier to PWM methods, is a clear separation between weak PPREs and those of medium and strong strength [29]. For the discovery of potential binding sites, this is extra information that could be especially of interest in processes considered context dependent, for example, for PPREs that reside in genomic context of transcription factor modules. Predicting the strength of PPAR binding can be a predictor of how prominent effect this receptor can have on a target gene. For example, if binding is easily competed by other transcription factors, the effect may not manifest in most tissues or it may manifest only in tissues expressing all transcription factors of a module containing the PPRE. As an example of the latter case, the *insulin-like growth factor binding protein 1* gene has a weak PPRE located inside a well-conserved area (suggesting presence of other transcription factor binding sites) and was only in liver responsive to PPAR ligands [59]. In contrast, genes with strong PPREs, such as *carnitine palmitoyltransferase* *1A* and *angiopoietin-like 4*, are PPAR responsive in many tissues (Heinäniemi et al., unpublished data).

## 7. CLUSTERING OF KNOWN PPAR TARGET GENES

**Figure 2:**
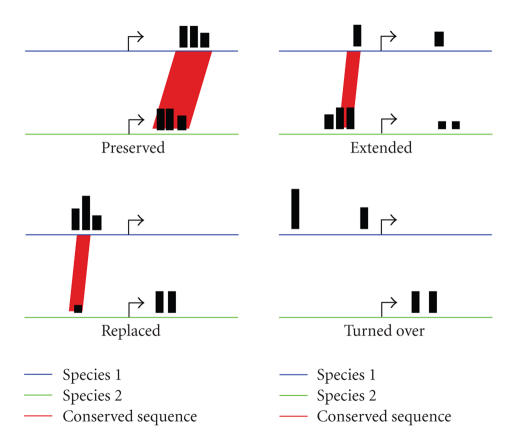
Possible evolutionary changes to PPRE location, strength, and conservation. Hypothetical genes from two different species (e.g., human and mouse) were compared for their PPREs (black bars, their height indicates relative strength). When the PPRE pattern is preserved, the genes will be sorted into cluster I, when extended in cluster II, when replaced in cluster III and when not at all conserved (e.g., when turned-over) in cluster IV.

The data added by binding strength analysis and by covering a larger regulatory region (±10 kB) was examined with all 38 human genes that are known to be primary PPAR targets together with their mouse ortholog. The clustering by predicted binding strength and evolutionary conservation of their PPREs resulted in four groups [29]. In general, clusters I to II contain genes that are well conserved between human and mouse. Cluster I contains genes that carry multiple conserved PPREs, while genes in cluster II have only one or two strong or medium conserved PPRE in human, which are found in comparable strength and location in the mouse. Cluster III contains genes that have strong or medium PPREs in one species that are conserved only as weak PPREs in the other species. Finally, cluster IV contains more than 25% of all tested genes, which have the common property that they carry one or more PPREs, but none of them is conserved. These examples suggest that regulation of target gene can survive turnover of binding sites and might even benefit from it as indicated in Figure 2.

The clustering analysis indicated some useful features for whole genome PPRE screens. Either the presence of at least one strong PPRE or more than two medium PPREs within the 20 kB surrounding the annotated TSS of a gene is a strong indication for a PPAR target gene. In this way, 28 out of the 38 the human genes would have been identified as PPAR targets. Similarly, for 29 of these 38 genes the analysis of their murine ortholog would have come to the same conclusion. A combination of these two criteria (passing the threshold in either the human or mouse ortholog) would have identified 37 of the 38 genes as PPAR targets.

### 7.1. A look at PPREs in their genomic context: putative target genes and binding modules

In the paper described above, the gene-dense human chromosome 19 (63.8 MB, 1445 known genes) and its syntenic mouse regions (956 genes have known orthologs) were selected for an in silico screening based on the above explained criteria; that is, both species were investigated for medium and strong PPREs (based on a PPAR*γ* prediction) [29]. Interestingly, 20% of genes of chromosome 19 contain a colocalizing strong PPRE and additional 4% have more than two medium PPREs or a proximal medium PPRE. These numbers suggest a total of 4000 to 5000 targets for PPARs in the human genome, if no false positives are assumed. Certainly, not all sites will be accessible and the human genome also contains weak binding sites that could gain function via interaction with other transcription factors. The latter can also be screened with the acquired knowledge on PPAR binding preferences down to 1% relative to the consensus PPRE. Experimentally, a complete evaluation of the selectivity of any such screen is complicated by the restricted expression profiles of the predicted genes, which prevents simple readouts from individual target tissues. When requiring the detection in human and mouse, 12.1% of genes from chromosome 19 were predicted as PPAR targets. In this approach, full alignment was not required, just preservation of what could be called PPAR binding potential. The more strong PPREs a gene has accumulated, the smaller the chances are that given all 250 human tissues none of these sites would get accessible or be built into a regulatory module with other transcription factor binding sites. Of relevance to cancer several cell cycle regulating genes were found by the screen, some of which have been reported as PPAR targets by others, such as *G1/S-specific cyclin E* [60], *p*19^*INK*4*d*^ [61], *prostate tumor overexpressed gene*, *serine protease hepsin* [62] and the serine/threonine kinases associated with cell cycle regulation *p21-activated kinase 4* (*PAK4*), and *homeodomain-interacting protein kinase 4*. In addition, the prostate tumor marker *kallikrein-3* [63] and several other *kallikrein* gene family members were detected. From novel targets, the regulatory regions of a ceramide synthesis regulator, *LASS1*, were experimentally confirmed [29]. Overexpression of this protein has been shown to restore normal ceramide levels and inhibit the growth of head and neck squamous carcinomas [64].

**Figure 3:**
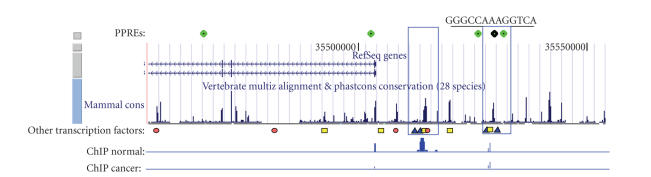
A gene module map compiled from bioinformatics data and experimental datasets. The superimposition of the PPRE track (in green on top) on other genome-wide datasets can reveal promising PPRE-containing binding modules for targeted therapy via PPAR activation. In this imaginary setting, transcription factor 1 (in blue) is known to be one main regulator of the hypothetical gene X and this regulation is altered in cancer. Transcription factor 2 (in yellow) synergistically activates gene X, but is lost in cancer cells. Chromatin immunoprecipitation data comparing normal and cancer binding profiles for this transcription factor reveal two main regulatory modules under normal conditions and a weaker binding in cancer samples due to loss of transcription factor 2. A colocalizing PPRE in module 2 could enable PPARs to replace transcription factor 2 in this module and to restore strong activation of this gene.

The complete list of putative PPAR target genes in chromosome 19 [29] offers interesting candidates representing physiological functions connected to PPARs. It will gain more power, when it can be integrated with other genomic screens, both experimental and bioinformatics, as has been outlined in the previous discussion. A vision for future of targeting cancer regulatory modules with colocalizing PPREs is depicted in Figure 3. A PPRE track (for simplicity binding strength was not indicated) provided by bioinformatics approaches can be compared against evidence of other regulatory modules provided by conservation analysis and screens for other transcription factors. Experimental data comparing regulation in a specific cancer type versus normal cells can be visualized in the same context to detect overlap in functional binding sites. Given the high interest of the scientific community to better characterize binding profiles of different transcription factors and the improved experimental techniques to detect genome-wide binding events, such additional tracks combined with a PPRE binding track could be available in near future.

## 8. CONCLUSION

The identification of genes showing a primary response to PPARs and their ligands, the so-called PPAR regulome, can be used as a prediction of their therapeutic potential as well as their possible side effects. Methods incorporating both experimental- and informatics-derived evidence to arrive at a more reliable prediction of PPAR targets and binding modules can bring all available data together with the aim to predict outcome in specific context. Taking the chromosome 19 in silico screening trial as an example and extrapolating the results to the whole human genome, we suggest that approximately 10% of all human genes (an estimate of 2000 to 2500 genes) have the potential to be directly regulated by PPARs by their PPRE content within 10 kB distance to their TSS. Translated to regulatory modules that colocalize with PPREs, an even larger number of genomic regions could be targeted by PPARs. In conclusion, in this review we have addressed the identification of direct targets using genomic sequences and binding data. In parallel, we have discussed the potential of looking for PPREs inside regulatory modules foreseeing that in future, very likely the emphasis will shift from target genes to target regulatory modules to alter a physiological response and from individual genes to whole genome response.

## LIST OF ABBREVIATIONS

DR1: Direct repeat spaced by one nucleotide
PPAR: Peroxisome proliferator-activated receptor
PPRE: PPAR response element
PWM: Position weight matrix
RXR: Retinoid X-receptor
TSS: Transcription start site
UCP3: Uncoupling protein 3.
